# Fendiline Enhances the Cytotoxic Effects of Therapeutic Agents on PDAC Cells by Inhibiting Tumor-Promoting Signaling Events: A Potential Strategy to Combat PDAC

**DOI:** 10.3390/ijms20102423

**Published:** 2019-05-16

**Authors:** Marwa Alhothali, Mevin Mathew, Geeta Iyer, Harshani R. Lawrence, Shengyu Yang, Srikumar Chellappan, Jaya Padmanabhan

**Affiliations:** 1 Department of Molecular Medicine, University of South Florida, 12901 Bruce B. Downs Blvd, Tampa, FL 33612, USA; marwa2@mail.usf.edu (M.A.); mevinm@mail.usf.edu (M.M.); iyerg@mail.usf.edu (G.I.); 2 Department of Tumor Biology, Moffitt Cancer Center and Research Institute, 12902 Magnolia Drive, Tampa, FL 33612, USA; sxy99@psu.edu (S.Y.); Srikumar.chellappan@moffitt.org (S.C.); 3 Department of Drug Discovery, Moffitt Cancer Center and Research Institute, 12902 Magnolia Drive, Tampa, FL 33612, USA; harshani.lawrence@moffitt.org; 4 Department of Cellular and Molecular Physiology, Penn State Cancer Institute, 400 University Drive, Hershey, PA 17033, USA; shengyuyang@gmail.com

**Keywords:** pancreatic ductal adenocarcinoma (PDAC), intracellular signaling, fendiline, visudyne, tivantinib, gemcitabine, cell cycle, anchorage independent growth, migration, self-renewal

## Abstract

The L-type calcium channel blocker fendiline has been shown to interfere with Ras-dependent signaling in K-Ras mutant cancer cells. Earlier studies from our lab had shown that treatment of pancreatic cancer cells with fendiline causes significant cytotoxicity and interferes with proliferation, survival, migration, invasion and anchorage independent growth. Currently there are no effective therapies to manage PDACs. As fendiline has been approved for treatment of patients with angina, we hypothesized that, if proven effective, combinatorial therapies using this agent would be easily translatable to clinic for testing in PDAC patients. Here we tested combinations of fendiline with gemcitabine, visudyne (a YAP1 inhibitor) or tivantinib (ARQ197, a c-Met inhibitor) for their effectiveness in overcoming growth and oncogenic characteristics of PDAC cells. The Hippo pathway component YAP1 has been shown to bypass K-Ras addiction, and allow tumor growth, in a Ras-null mouse model. Similarly, c-Met expression has been associated with poor prognosis and metastasis in PDAC patients. Our results presented here show that combinations of fendiline with these inhibitors show enhanced anti-tumor activity in Panc1, MiaPaCa2 and CD18/HPAF PDAC cells, as evident from the reduced viability, migration, anchorage-independent growth and self-renewal. Biochemical analysis shows that these agents interfere with various signaling cascades such as the activation of Akt and ERK, as well as the expression of c-Myc and CD44 that are altered in PDACs. These results imply that inclusion of fendiline may improve the efficacy of various chemotherapeutic agents that could potentially benefit PDAC patients.

## 1. Introduction

Pancreatic Ductal Adenocarcinoma (PDAC) is the fourth leading cause of cancer related deaths in the world. In 2016, it became the third leading cause of cancer deaths in the United States and it is expected to become the second leading cause by 2030 [1,2]. It is considered one of the deadliest and most aggressive cancers, with a 5-year survival rate of only 7% [3,4]. Approximately 30% of PDAC patients exhibit locally advanced disease and more than 50% have metastases at distant locations at the time of diagnosis [5,6]. Therefore, surgical resection is beneficial to only a minority of patients (10–20%), who are diagnosed prior to metastasis. Surgical resection increases the long-term survival, however, recurrence still occurs post-surgery in almost all patients and chemotherapy combined with radiation is the treatment option for such patients. The chemotherapeutic agent gemcitabine, alone or in combination with other agents, had been the first line of treatment for both locally advanced and metastatic pancreatic cancer [7]. The response rate to gemcitabine is still minimal and most patients develop resistance, which is a major hurdle in pancreatic cancer therapy [8,9]. Though treatment with FOLFIRINOX and gemcitabine/nab-Paclitaxel combination improves survival, long-term survival is still poor and additional therapies are being sought out to effectively manage PDACs [5,10,11].

The majority of the PDAC patients show activating mutations in K-Ras, which locks it in the GTP-bound state, enabling continuous activation of downstream signaling pathways, including the MAPK, PI3K, and RalGDS cascades [12,13,14,15,16,17,18]. RalGEF is a downstream effector of Ras that activates the Ras family proteins RalA and RalB, which are known to play a role in tumor growth and metastasis; RalA is involved in tumor initiation, whereas RalB is known to promote tumor metastasis [19,20,21]. In addition to oncogenic Ras, inactivation of CDKN2A (p16, cyclin-dependent kinase inhibitor 2A) is observed in approximately 90% of PDAC cases [22]. Similarly, PDAC patients show overexpression of inhibitor of differentiation, Id1, a helix-loop-helix (HLH) protein, which correlated with tumor angiogenesis and a low survival rate [23]. Double knockdown of Id1/Id3 has been shown to reduce the metastatic potential in preclinical models of pancreatic cancer, suggesting that treatments that reduce the expression of these proteins would be beneficial for the PDAC patients [24]. Similarly, activation of Src, a non-receptor tyrosine kinase, has been observed in many cancers including that of pancreas, and an inhibition of the Src signaling axis has been associated with inhibition of stemness and oncogenic potential [25]. Furthermore, studies in PDAC cells have shown that activated Src can induce Id1 expression [26], thus suggesting that inhibition of Src signaling would interfere with the Id1-associated tumor growth as well.

Another major pathway that is deregulated in PDACs is the Hippo signaling cascade [27,28,29]. Hippo pathway is involved in control of organ size, cell proliferation, stem cell activity, regeneration, and tumor suppression [30,31,32,33]. It functions mainly by downregulating its downstream target YAP1 (YES-associated protein 1) and the related TAZ. YAP1 is a transcriptional co-activator, which upon activation can promote cell proliferation and suppress apoptosis, and has been shown to play a role in tumorigenesis, including that of pancreas [27,28,29]. Previous studies on mouse models of PDACs have shown that YAP1 promotes the growth of PDACs in a Ras-null model [34]. The study performed in a dox-inducible *K-Ras*^G12D^ GEM PDAC mouse model showed that extinction of K-RasG12D can completely regress tumor growth. However, the tumor relapsed in 70% of the mice in approximately two months with a more aggressive phenotype, and these K-Ras-relapsed tumors showed amplification of YAP1. Studies have also shown that YAP1 plays an important role in the neoplastic transformation and progression of K-Ras mutant pancreatic ductal cells into invasive PDACs [35,36]. These results imply that co-treatment of PDAC cells with YAP1 and Ras inhibitors could potentially overcome the growth and metastasis of PDACs. 

Targeting Ras using small molecule inhibitors have been challenging. Studies by Hancock and colleagues have shown that the L-type calcium channel blocker fendiline specifically elicits cytotoxicity in K-Ras mutant cancer cells by interfering with K-Ras localization at the membrane [37,38,39]. Our published studies showed that fendiline significantly inhibits proliferation, survival and metastatic characteristics of PDAC cells, as evident by reduced migration, invasion and anchorage independent growth [40]. In the current study we tested if combining fendiline with the YAP1 inhibitor visudyne (Verteporfin), which is used for photodynamic therapy of macular degeneration, [41,42,43,44,45,46], elicits additive inhibitory effects on survival and metastatic characteristics of PDAC cells. Additionally, experiments were also conducted with combinations of fendiline and gemcitabine, the nucleoside analog commonly used to treat PDAC. Efficacy of combining fendiline with tivantinib (ARQ987), a c-Met inhibitor, was also tested, since c-Met overexpression has been associated with poor prognosis in PDAC patients; c-Met-targeted therapies are in clinical trial for various solid tumors [47,48,49,50,51,52]. Our data suggest that addition of fendiline enhances the efficacy of visudyne, tivantinib, or gemcitabine and reduces survival, stemness, and metastatic properties of PDAC cells.

## 2. Results

### 2.1. Combinatorial Treatment with Fendiline Shows Enhanced Cytotoxic Effects on PDAC Cells

**Figure 1:**
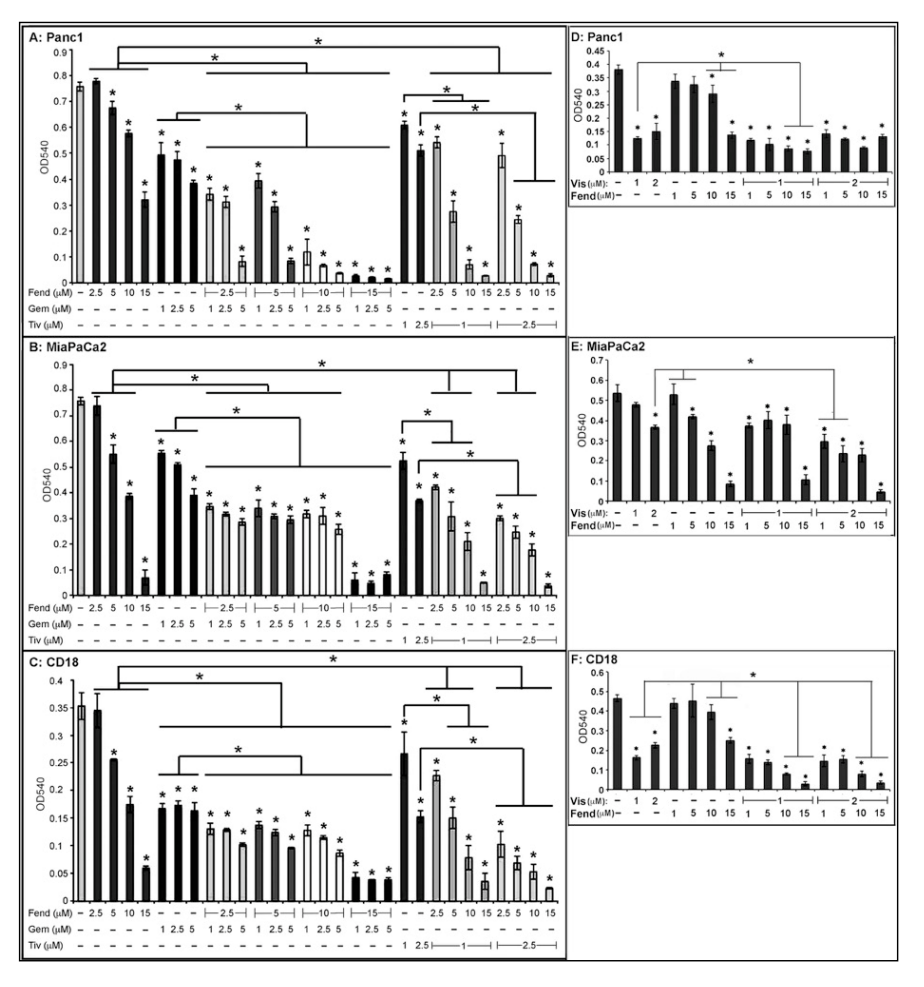
Fendiline significantly inhibits viability of PDAC cells when combined with visudyne, gemcitabine or tivantinib: Panc1 (**A**,**D**), MiaPaCa2 (**B**,**E**) or CD18 (**C**,**F**) cells were treated with indicated concentrations of fendiline (Fend: 1 µM, 2.5 µM, 5 µM, 10 µM and 15 µM), gemcitabine (1 µM, 2.5 µM and 5 µM), tivantinib (1 µM and 2.5 µM), or visudyne (1 µM and 2 µM), alone or in combination, and viability was measured after 48 h by MTT assays. Results show that the tested concentrations of fendiline and the chemotherapeutic agents show differential effects on the PDAC cells; MiaPaCa2 cells were very sensitive to high concentrations of fendiline by itself (**B**). Panc1 cells were more sensitive to gemcitabine in combination with fendiline and MiaPaCa2 were least sensitive (A vs. B). Similarly, tivantinib in combination with fendiline showed marked inhibition of viability in all the cells (**A**–**C**). Visudyne showed strong inhibition of viability of Panc1 and CD18 cells as a single agent and the inhibition was significantly more in combination with fendiline at 10 and 15 µM (**D**–**F**). Experiments were conducted in triplicates and were repeated at least 3 times with different combinations of the drugs. Statistical analysis was conducted between vehicle treated and drug treated samples as well as single agent vs. combinatorial treatment; statistically significant differences are denoted by * (* *P* < 0.05).

Studies on pancreatic cancer cells have shown that fendiline elicits strong growth inhibition and cytotoxic effects. Here we conducted studies using Panc1, MiaPaCa2 and CD18/HPAF (referred to as CD18) cells to determine if combinations of fendiline with the YAP inhibitor visudyne, c-met inhibitor tivantinib or the commonly used PDAC drug gemcitabine elicit additive inhibition on viability. Both Panc1 and MiaPaCa2 cells are mesenchymal in nature and CD18 cells elicit more epithelial characteristics. These cells were treated with indicated concentrations of gemcitabine (1 µM, 2.5 µM or 5 µM), tivantinib (1 µM or 2.5 µM), or visudyne (1 µM or 2 µM), alone or in combination with fendiline (1 µM, 2.5 µM, 5 µM, 10 µM or 15 µM) for 48 h and cell viability was assessed by MTT assay [40,53]. The results presented in Figure 1 A–C show that the combinatorial treatment with fendiline and gemcitabine or tivantinib show stronger growth inhibitory effects when compared to single agent treatment, with some variation in response between the cell lines. The inhibition was clearly evident in Panc1 and CD18 cells when fendiline was combined with tivantinib or gemcitabine at all the concentrations tested (Figure 1A,C). MiaPaCa2 cells were very sensitive to the treatment with 15 μM fendiline alone and addition of gemcitabine or tivantinib at this fendiline concentration did not show any further reduction in viability, but lower concentrations of fendiline showed an additive effect with gemcitabine and tivantinib in these cells as well (Figure 1B). Treatment with visudyne, fendiline or a combination of these two drugs showed strong inhibition of viability at higher concentrations of fendiline (10 and 15 µM) in Panc1 and CD18 cells (Figure 1D,F); MiaPaCa2 cells showed additive effects at lower concentrations of fendiline and as mentioned earlier, fendiline alone at 15 µM significantly inhibited the viability of these cells (Figure 1E). These results suggest that fendiline could potentially enhance the efficacy of established chemotherapeutic agents, but the magnitude of these effects may vary between the drugs.

Cells treated with tivantinib alone or in combination with fendiline showed morphological changes that are reminiscent of mitotic arrest and apoptosis; these effects were more pronounced in MiaPaCa2 and Panc1 cells (Supplementary Figure S1). The other agents showed a reduction in growth but not many apoptotic cells were visualized after 24 h of treatment. Supporting this observation, treatment with tivantinib resulted in a high number of TUNEL positive cells, which was drastically increased upon combined treatment with fendiline (Supplementary Figure S2).

**Figure 2:**
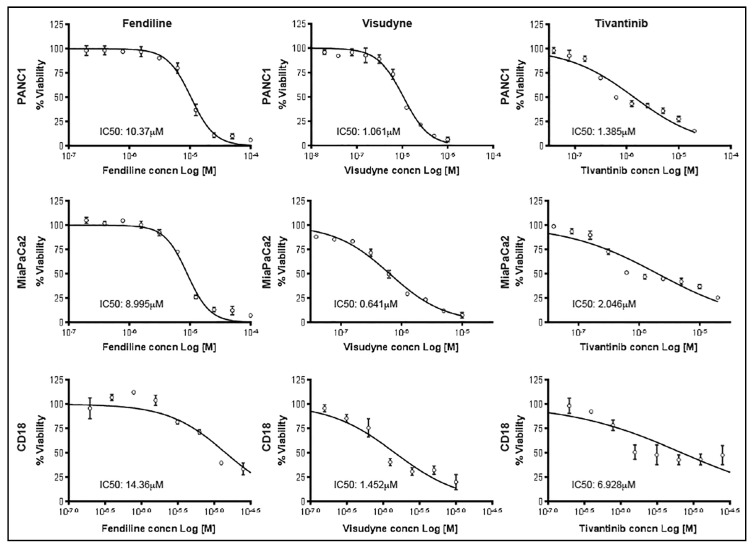
IC50 determination in PDAC cells: PDAC cells treated with incremental concentrations of fendiline, visudyne or tivantinib and viability was determined by MTT assay after 48 h. IC_50_ values were determined using GraphPad Prism 6 software.

To determine the IC_50_ values of tivantinib and visudyne on the PDAC cells, we conducted a viability assay after treatment of the cells with incremental concentrations of the drugs. As can be seen from the results presented in Figure 2, MiaPaCa2 cells were most sensitive to fendiline and visudyne followed by Panc1; CD18 cells appeared to be the least sensitive to the treatments but all these cells responded well to the growth inhibitory effect upon combinatorial treatment.

### 2.2. Treatment with Fendiline and the Pharmacological Agents Reduce Migration of PDAC Cells 

**Figure 3:**
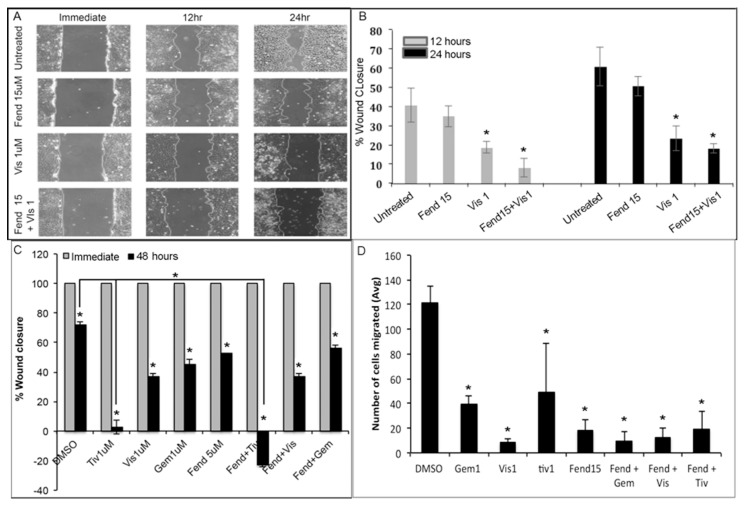
Panc1 cells treated with combination of visudyne and fendiline show significantly lower rate of migration: (**A**,**B**) Scratch wound was made in monolayers of Panc1 cells and the wound were allowed to close in the presence or absence of visudyne 1 µM (Vis 1), fendiline 15 µM (Fend 15) or a combination of these 2 drugs. Wound area was measured immediately and after 12 and 24 h of treatment and wound closure was calculated from the initial wound area. Results show that combinatorial treatment significantly reduces the migration of the cells at 12 and 24 h. A: Magnification 10×. (**C**) Wound healing in Panc1 cells treated with or without Fendiline (5 µM), Tivantinib (1 µM) (Tiv 1), Visudyne (1 µM) or Gemcitabine (1 µM) (Gem 1) alone or in combination analyzed after 48 hours of treatment shows that the combination of Fendiline and Tivantinib not only inhibits migration but also induces apoptosis in the cells as the wound area was significantly increased after 48 h with the combination. Combining the other two drugs with Fendiline at 5 µM did not show any additive effects. * *P* < 0.05. (**D**) Panc1 cells treated with the indicated drugs show reduced invasion in the presence of the drugs alone and in combination with fendiline.

The combinatorial treatment showed strong inhibitory effects on the growth of the PDAC cells we examined if the different treatments interfered with various biological properties, such as migration, invasion, anchorage independent growth and self-renewal, of the cancer cells. Though we tested both Panc1 and MiaPaCa2 cells for migratory changes, since MiaPaCa2 cells showed increased cell death and detachment upon reaching confluence, Panc1 was mainly used for analysis. Cells were treated with 15 µM Fendiline, 1 µM Visudyne, or a combination of these two drugs and the wound area was measured immediately as well as 12 and 24 h after making the wound. Results showed that these drugs reduce the migration when used individually but the effect was more prominent when combined (Figure 3A,B). Next, we compared the migration in the presence of a lower dose of fendiline (5 µM) alone or together with gemcitabine, visudyne or tivantinib at 1 μM concentration. Results showed that fendiline together with tivantinib at these lower concentrations inhibit migration and induce cell cycle arrest and apoptosis at 48 h (Figure 3C). Neither visudyne nor gemcitabine showed any additive effects in the presence of 5 µM fendiline, suggesting that higher concentration of fendiline is required to see additive effects on migration with these drugs. These data further support the notion that combinatorial treatment with fendiline may effectively interfere with growth and metastatic characteristics of PDAC cells, but the concentration required us to see if an additive effect differs and may depend on the therapeutic agent being used in combination. We also conducted an analysis of the invasive property of the cells using Boyden chambers. Pretreated Panc1 cells were plated into the transwell chamber with or without the drugs, incubated and the invaded cells were counted after 5 h. Results presented in Figure 3D shows inhibition of invasion of cells upon treatment with single agent or in combination. Visudyne was the most effective and tivantinib was the least. Fendiline in general showed strong inhibition of invasion.

### 2.3. Co-Treatment with Fendiline and the Pharmacological Agents Inhibits Anchorage Independent Growth of PDAC Cells 

**Figure 4:**
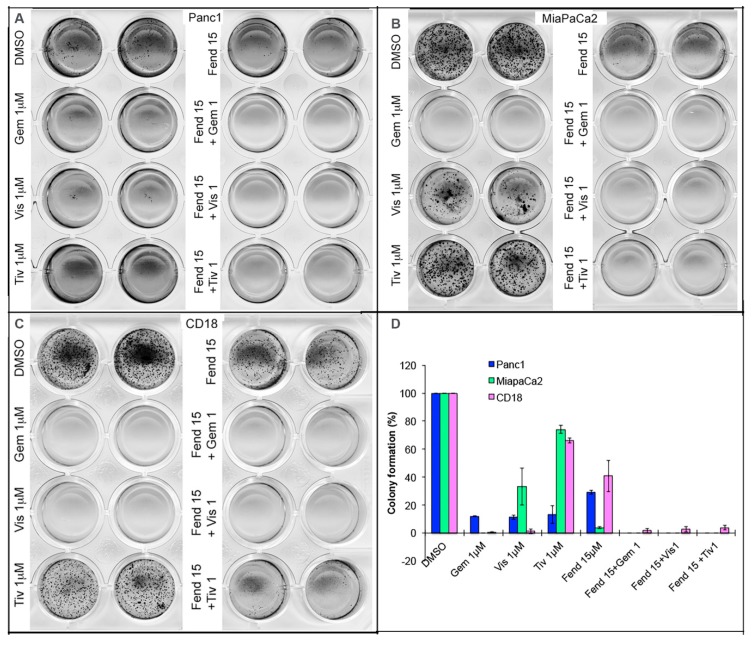
Anchorage independent growth of PDAC cells is inhibited by combinatorial treatment with fendiline: 2500 Panc1 (**A**), MiaPaCa2 (**B**) or CD18 (**C**) cells were allowed to form colonies on soft agar, in the presence or absence of 1 μM gemcitabine, visudyne or tivantinib, alone or in combination with 15 µM fendiline. Results show cell and agent-specific reduction in colony formation in cells treated with individual agents and almost complete inhibition in the wells treated with the drug combination. Solo gemcitabine treatment strongly inhibited colony formation in both CD18 and MiaPaCa2 cells. Tivantinib treatment resulted in a significant reduction in colony formation in both CD18 (**C**) and MiaPaCa2 (**B**) cells and co-treatment with 15 µM fendiline almost completely eliminated colony formation; (**D**) quantification of the colonies from different treatment, which shows almost complete inhibition with all the agents under combinatorial treatment.

To determine if treatment with fendiline and the combinations of agents mentioned here affects anchorage independent growth of PDAC cells, we conducted soft agar colony formation assay. Cells were seeded in the presence of drug or vehicle and were replenished with drug containing medium once a week. Colonies were allowed to form over a period of 2–3 weeks and were analyzed after MTT staining. We observed that co-treatment with fendiline and gemcitabine, visudyne or tivantinib drastically reduced soft agar colony formation (Figure 4A–C) compared to single agent treatment in the PDAC cells. Gemcitabine was very effective as a single agent in eliminating colony formation in both CD18 and MiaPaCa2 cells; visudyne was most effective in inhibiting colony formation in CD18 cells. Tivantinib at 1 µM showed a significant but comparably lower inhibition in colony formation in CD18 and MiaPaCa2 cells compared to Panc1. Fendiline at 15 µM showed a very strong inhibition on colony formation in MiaPaCa2 compared to Panc1 and CD18 cells, but when tivantinib was combined with fendiline, the effect was much stronger and colony formation was almost completely eliminated in all the cell lines. Generally, Panc1 cells formed fewer colonies than both CD18 and MiaPaCa2 cells when plated at the same density. Though these results show cell-line dependent differences, all of them responded well to the combinatorial treatment, implying that fendiline might enhance the therapeutic potential of established and developing chemotherapeutic agents. Adherent clonogenic assay conducted on plastic also showed that treatment with tivantinib, visudyne or fendiline drastically inhibit colony formation in a concentration dependent manner and the combination eliminated colony formation (data not shown). 

### 2.4. Self-Renewal of PDAC Cells is Drastically Reduced upon Treatment with Single Agents and Completely Eliminated by Combinations of the Drugs

**Figure 5:**
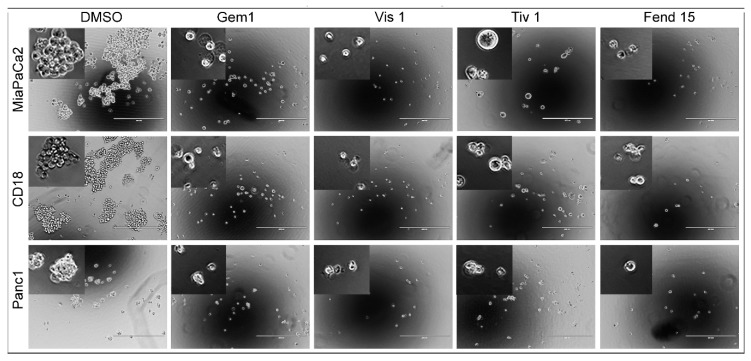
PDAC cells treated with fendiline, gemcitabine, visudyne or tivantinib show reduced sphere formation: MiaPaCa2, CD18 or Panc1 cells were plated with or without 1 μM Gemcitabine (Gem1), 1 µM visudyne (Vis 1), 1 µM tivantinib (Tiv 1) or 15 µM fendiline (Fend 15) alone or in combination (combination is not shown as the self renewal was completely inhibited by the treatment). Spheres were analyzed 10 days after plating using an Evos inverted microscope. Maximum effect was observed with visudyne followed by fendiline in all the cells. The inset shows magnified image of the spheres from each treatment. Scale bar shown is 400 µm; magnification 4×.

YAP1 has been associated with stemness in cancer cells and its inhibition has been shown to interfere with growth of stem-like cells [54,55]. Here we conducted sphere formation assays to examine the effects of gemcitabine, visudyne or tivantinib as single agents or in combination with fendiline on self-renewal capacity [55]. MiaPaCa2, CD18 or Panc1 cells were grown in serum free stem-cell selective medium on ultra low attachment 96 well plates. As presented in Figure 5, all the treatments drastically decreased the sphere formation in all the cells. 1 μM visudyne alone completely eliminated the self-renewal of the PDAC cells, implying that these drugs can act independently to interfere with the growth of stem-like cells. Fendiline at 15 µM completely eliminated self-renewal of the MiaPaCa2 cells while Panc1 and CD18 appeared to have very few smaller spheres. Both gemcitabine and tivantinib eliminated self-renewal of the PDAC cells when combined with fendiline. 

### 2.5. Comparative Analysis of Tivantinib, Gemcitabine and Visudyne on PDAC Cells Show Differential and Co-Operative Effects of the Drugs on Proteins Involved in Oncogenic Signaling

**Figure 6:**
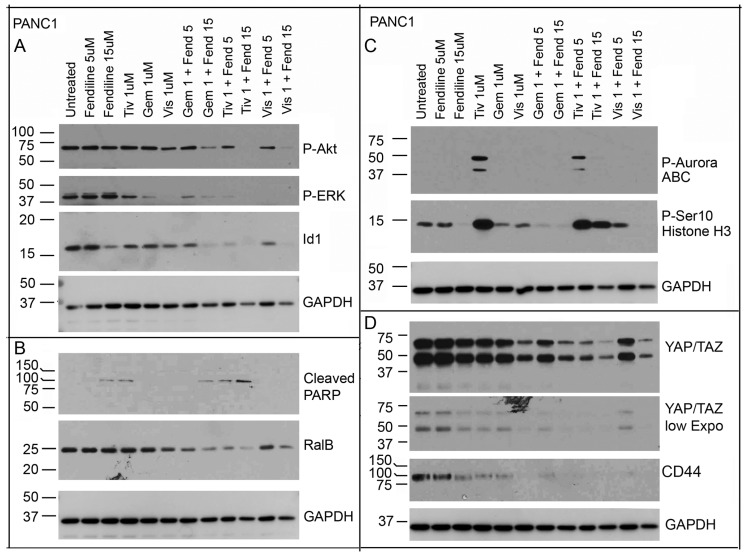
Panc1 cells treated with fendiline alone or along with tivantinib, gemcitabine or visudyne show differential effects on proteins involved in tumor cell survival, proliferation and mitosis: Panc1 cells were treated with 1 μM tivantinib, gemcitabine or visudyne alone or along with fendiline at 5 µM or 15 µM and western blot analysis was performed with the indicated antibodies. (**A**,**B**) Results show that the combinatorial treatment strongly inhibits Akt and ERK activation as well as Id1 and RalB expression. All these changes were most visible in samples from cells treated with a combination of 15 µM fendiline and the drugs. Analysis of the blots using antibodies against cleaved PARP showed that fendiline at 15 µM and tivantinib treatment elicit PARP cleavage, which was increased in the samples from the co-treatment, indicative of increased apoptosis. Blots were reprobed with GAPDH antibodies for protein normalization. (**C**) Tivantinib treatment resulted in a significant increase in Aurora ABC as well as Histone H3 phosphorylation whereas gemcitabine or visudyne showed no changes; fendiline at 15 μM inhibited Histone H3 phosphorylation but had no effect on the tivantinib-mediated H3 phosphorylation. Tivntinib-mediated Aurora kinase phosphorylation was inhibited upon co-treatment with fendiline. Blots were reprobed with GAPDH antibody for protein normalization. (**D**) Analysis of the blots using YAP/TAZ antibodies showed that visudyne by itself strongly inhibits both YAP and TAZ. Fendiline (15 µM) together with tivantinib, gemcitabine or visudyne showed a marked reduction in the levels of these proteins. Treatment with single agents also appeared to inhibit the expression of YAP-TAZ to some extent (evident on the lower exposure). Blots were reprobed with GAPDH antibody for protein normalization.

Our results above showed that gemcitabine, visudyne and tivantinib elicit strong growth inhibitory effects and interfere with migration, anchorage independent growth and self-renewal of PDAC cells. To determine the potential mechanisms by which these agents interfere with growth and tumor-associated signaling, we conducted western blot analysis on samples from cells treated with the various combinations of drugs. An analysis of Panc1 cells, treated with fendiline (5 µm and 15 µM) alone or in combination with tivantinib (1 µM), visudyne (1 µM) or gemcitabine (1 µM) for 24 h, showed that combination of the various drugs with 15 µM fendiline effectively inhibit both Akt and ERK activation (Figure 6A). In the case of ERK, the phosphorylation was reduced upon treatment with tivantinib, visudyne or gemcitabine as single agents as well, but the effect was more pronounced in the combinatorial treatment (Figure 6A, panel 2). The same blots analyzed using an Id1 antibody showed that its levels were also significantly reduced upon treatment with the combinations as well as by fendiline itself; among the treatments, tivantinib appeared to elicit the maximum effect when combined with fendiline (Figure 6A, panel 3). We analyzed the samples using cleaved PARP antibody to determine if the cells are undergoing apoptosis upon treatment. Samples from 15 µM fendiline and 1 µM tivantinib treated cells showed cleaved PARP and its levels were increased upon cotreatment with these drugs, at 15 µM fendiline concentration (Figure 6B). A combination of gemcitabine with fendiline also showed PARP cleavage but visudyne treated samples did not show any cleaved PARP at the concentrations and durations tested. Since RalB has been implicated in PDAC invasion and metastasis and is known to be one of the most significantly upregulated genes in patient-derived metastasis we examined if the expression of this Ras family member is affected upon treatment. Analysis of the membranes with RalB antibodies, showed that combinatorial treatment with gemcitabine, tivantinib or visudyne with 15 µM fendiline efficiently reduced its levels (Figure 6B). RalA also showed a slight reduction in the levels but the effect was much less pronounced than that observed with RalB (data not shown). 

Since the PDAC cells treated with tivantinib appeared to undergo morphological changes resembling mitotic arrest (Supplementary Figure S2), we examined for changes in Phospho-Histone H3 and Phospho-Aurora Kinase ABC in the treated cells. The data presented in Figure 6C shows that samples from tivantinib treated cells show increased levels of Histone H3 and Aurora kinase phosphorylation indicative of mitotic arrest (Figure 6C, panel 1 and 2). These results agree with earlier reports, which showed that tivantinib elicits c-Met-independent cytotoxic effects and induces microtubule depolymerization and H3 phosphorylation [56,57,58,59,60,61]. Fendiline co-treatment inhibited tivantinib-mediated increase in P-Aurora Kinase. Cells treated with fendiline alone showed inhibition of Histone H3 phosphorylation but had very little effect on the tivantinib-mediated H3 phosphorylation (Figure 6C, panel 1 and 2). Our published studies have shown that fendiline induces G1 arrest in PDAC cells [40] and the decrease in H3 phosphorylation in cells treated with this agent agrees with the previous results. Treatment with gemcitabine or visudyne as single agents showed no significant effect on phosphorylation of Histone H3 or Aurora kinase; gemcitabine when combined with fendiline inhibited the basal levels of histone H3 phosphorylation observed in the vehicle treated Panc1 cells, suggestive of inhibition of mitotic progression. The blots were re-probed with GAPDH antibody for protein normalization. 

Since visudyne is a known YAP1 inhibitor, we next examined if YAP1 levels were altered under the various treatments. Examination of the blots using YAP/TAZ antibodies showed a reduction in the levels of these proteins in cells treated with visudyne; treatment with tivantinib or gemcitabine alone did not affect the levels of YAP1, but combinatorial treatment with fendiline showed a clear reduction in both YAP and Taz proteins (Figure 6D, top two panels show different exposures of the same blot). These data suggest that when combined with fendiline both gemcitabine and tivantinib would be as effective as visudyne in inhibiting YAP-dependent signaling in PDACs. More importantly, since the combinatorial treatment showed inhibition of YAP1, we expect these drugs to show efficacy in K-Ras-independent PDAC tumors, where YAP1 is known to compromise for the loss of K-Ras to promote tumor growth. Though we expected visudyne to elicit the maximum effect, the results showed that Tivantinib and fendiline show stronger inhibition on YAP and Taz. The reason for this is unclear at this point. It is possible that higher concentrations or longer treatment with visudyne is necessary to see better inhibition of YAP and Taz in these cells. Studies in Panc1 cells have shown that visudyne induces PARP cleavage and reduces YAP levels at concentrations ranging from 2 to 8 µM [62]. Furthermore, studies by others have shown that paracrine signaling by HGF/c-MET enhances YAP nuclear localization and activation in PDAC cells, and it is possible that tivantinib interferes with the nuclear translocation of YAP, leading to its cytoplasmic retention and degradation [63]. 

Since the treatment with the various agents interfered with self-renewal of the cancer cells we expected that markers of stemness might be affected by the treatment. Though we tested for changes in Sox2 or Oct4, we were unable to detect these by western blotting are affected. Studies in PDACs have shown that high expression of CD44 correlates with presence of cancer stem cells (CSC) [64,65]. Our published studies have shown that both MiaPaCa2 and Panc1 express high levels of CD44 and the expression is reduced upon treatment with fendiline [40]. An analysis of the blots using the CD44 antibody showed a reduction in its levels in Panc1 cells treated with single agents and with combinations of fendiline (Figure 6D); CD44 is a cell surface receptor, also known as a hyaluroran receptor, and its expression correlates with adhesion, migration and chemoresistance in PDACs [66]. Furthermore, the expression of this cell surface protein is associated with poor prognosis and its downregulation has been associated with inhibition of tumor growth. Our results from the combinatorial treatment with fendiline on the PDAC cells suggest that fendiline would be able to overcome CD44-associated oncogenic signaling in PDACs.

**Figure 7:**
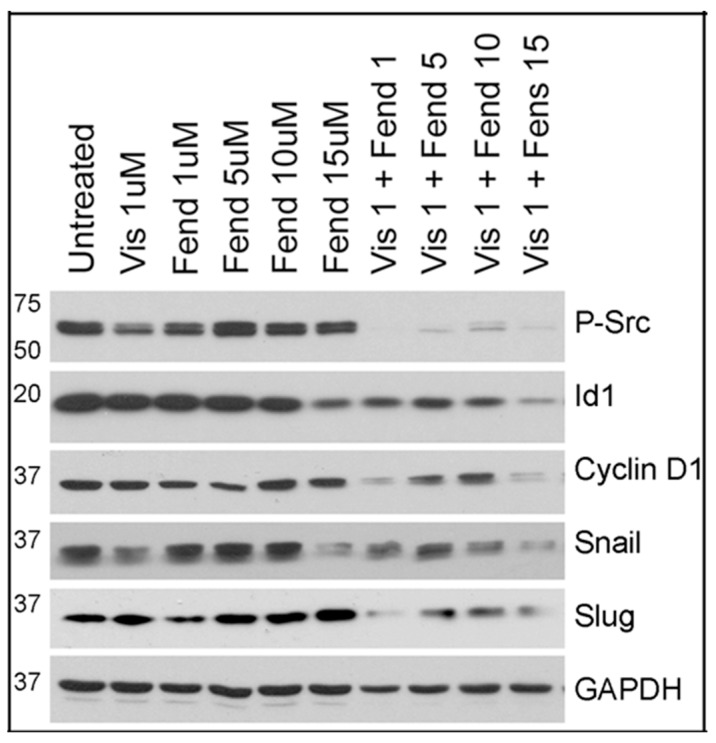
Panc1 cells treated with visudyne and varying concentrations of fendiline show additive effect on inhibition of oncogenic signaling: Samples from Panc1 cells treated with indicated concentrations of fendiline or visudyne alone or in combination were analyzed using phospho-Src, Id1, Cyclin D1, Snail or Slug antibodies by western blotting. Results show that combinatorial treatment reduces the levels of all these proteins even at the lowest concentration of fendiline. GAPDH antibody was used to reprobe the blot for protein normalization.

Furthermore, to test if lower concentrations of fendiline bring about strong anti-oncogenic signaling we treated Panc1 cells after treatment with varying concentrations of fendiline alone or together with visudyne (1 µM). Results presented in Figure 7 show strong inhibition of Phospho-Src, Id1, Cyclin D1, Snail and Slug even with the lowest concentration of fendiline and visudyne that we tested here (Figure 7). Src activation is associated with tumor progression, invasion, metastasis as well as therapy resistance in PDACs [67,68] and it is known to induce Id1 expression. Therefore, an inhibition of this signaling cascade would be ideal when considering treatment strategies for the PDAC patients. Slug and snail are associated with epithelial mesenchymal transition (EMT) and these transcription factors are overexpressed in PDACs, where their expression has been correlated with the grade of the tumor [69,70]. Inhibition of Slug and Snail suggest that combinatorial treatment with fendiline and a YAP inhibitor would potentially interfere with EMT and metastasis in PDACs. 

**Figure 8:**
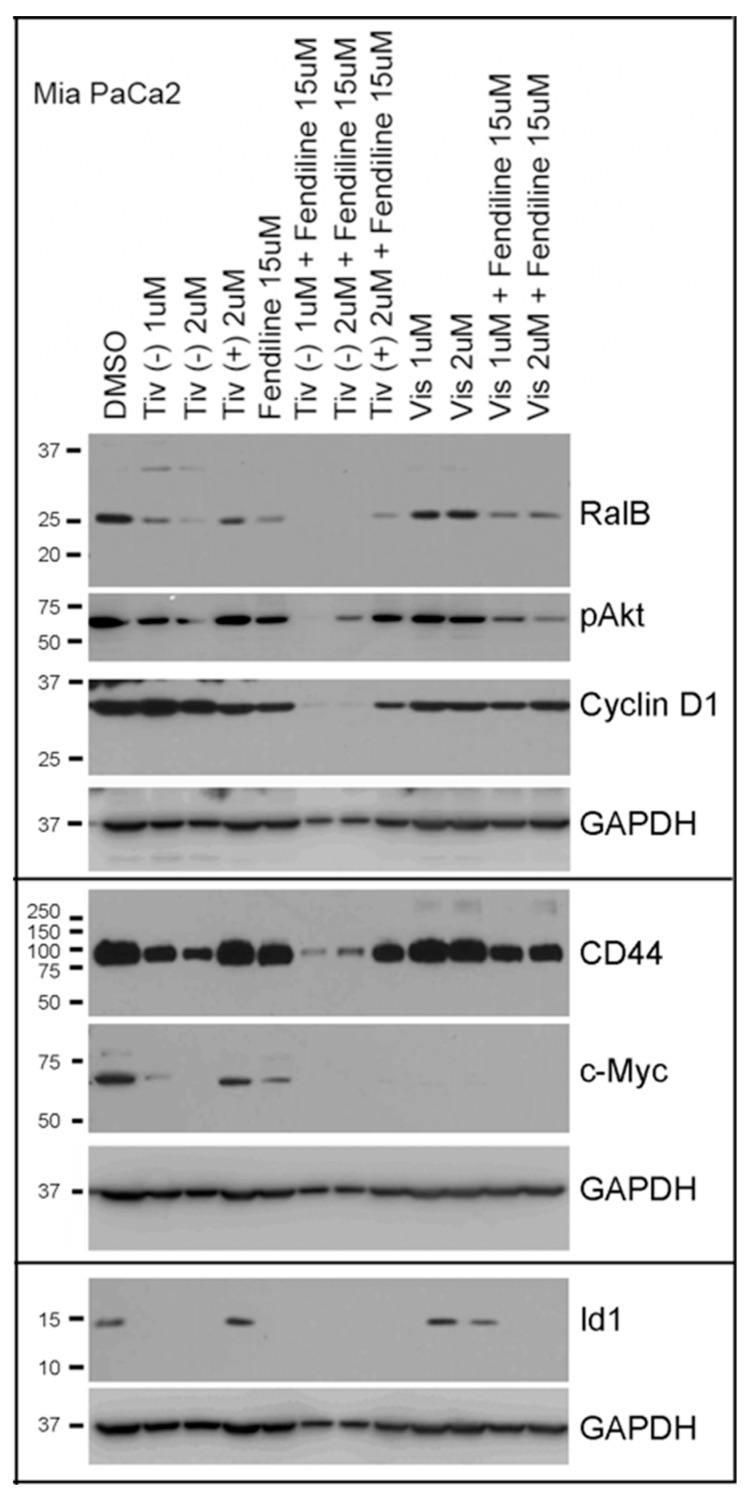
MiaPaCa2 cells treated with a combination of tivantinib or visudyne and fendiline show stronger inhibition of oncgenic signaling: Cells were treated for 24 h with the indicated concentrations of drugs and samples were analyzed by PAGE and western blotting using the antibodies shown. Tivantinib in combination with fendiline showed strong inhibition of CD44, c-Myc, RalB, Cyclin D1, Id1 and P-Akt, implying some of the potential tumor-associated signaling mechanisms targeted by these agents.

Results similar to that observed with Panc1 cells were also obtained with MiaPaCa2 cells treated with varying concentrations of the drugs (Figure 8). The Chemistry core at Moffitt had supplied us with the inactive enantiomer of tivantinib (Tiv (+)) [61], which is included in the data shown here; Tiv (−) is same as ARQ197. The inactive tivantinib behaved very similar to the DMSO vehicle control in viability analysis conducted on the PDAC cells (data not shown). The active tivantinib as a single agent showed strong inhibition of various tumor-promoting signaling molecules, such as reduced RalB, CD44, c-Myc, and Id1 as well as inhibition of Akt activation, whereas the inactive enantiomer did not elicit much effect (Figure 8). Fendiline treatment alone resulted in a reduction in RalB, Phospho-Akt, c-Myc and Id1 and when combined with tivantinib the effects were stronger (Figure 8). Visudyne also showed better effects when combined with fendiline, implying that combination of the drugs with fendiline would be better for overcoming growth and metastatic properties associated with PDAC cells. Previous studies from our lab had shown that fendiline inhibits c-Myc expression in PDAC cells thereby interfering with Myc-dependent oncogenic signaling [40]. Blots reprobed with GAPDH antibody was used for protein normalization.

### 2.6. CD18 Cells Treated with Fendiline alone or along with Tivantinib, Gemcitabine or Visudyne Show Additive Effects on Inhibition of Id1

**Figure 9:**
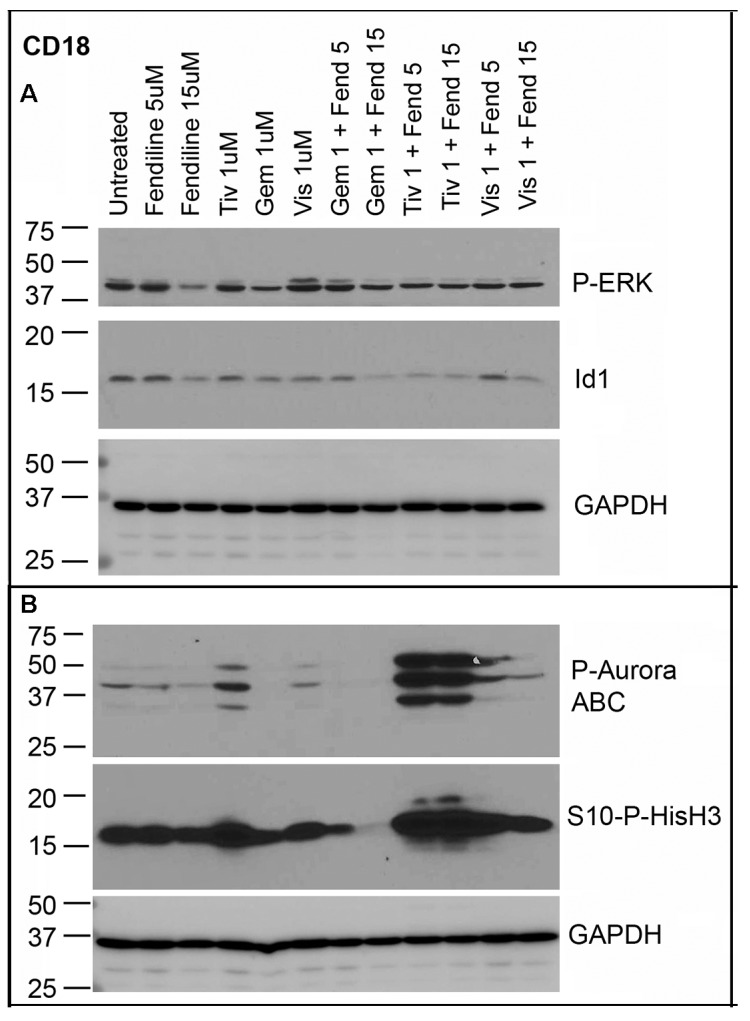
CD18 cells treated with fendiline alone and in combinations with the pharmacological agents show altered expression of proteins associated with proliferation, differentiation and cell cycle: CD18 cells treated with or without 5 µM and 15 µM fendiline alone or along with 1 µM of tivantinib, gemcitabine or visudyne showed decreased Id1 expression (**A**), which was more prominent after co-treatment. Blots were reprobed with GAPDH for normalization of proteins. (**B**) Cells treated with tivantinib showed an increase in the levels of Phospho-Aurora ABC and Phospho-Histone H3, which were further increased upon co-treatment with fendiline. Blots were reprobed with GAPDH antibody for protein normalization.

As mentioned earlier, Panc1 and MiaPaCa2 cells exhibit more mesenchymal characteristics whereas CD18 cells are more epithelial in nature. To determine if the drugs show similar effects as in the case of the mesenchymal cells, we treated CD18 cells with fendiline at 5 µM and 15 µM concentrations alone or along with 1 µM tivantinib, gemcitabine or visudyne for 24 h and examined for changes in the expression of some of the above mentioned proteins. Analysis of Phospho-ERK showed that treatment with 15 µM fendiline reduces its levels in CD18 cells but in combination with the other agents did not show any further decrease (Figure 9A, top panel). Analysis of the blots using an Id1 antibody showed that treatment with 15 µM fendiline by itself shows a decrease, which is further reduced upon co-treatment with the various agents (Figure 9A, panel 2). We were unable to detect any slug, snail or CD44 in these cells. Analysis of the blots with phospho-Histone H3 and phospho-Aurora kinase ABC antibodies showed a marked increase in their levels in tivantinib treated cells; unlike the results from Panc1 cells, levels of these phospho-proteins were further increased in samples co-treated with fendiline and tivantinib (Figure 9B, top two panels). Gemcitabine on the other hand as a single agent and upon co-treatment with fendiline showed a reduction in the levels of both phospho-Histone H3 and phospho-Aurora ABC; gemcitabine has been shown to induce S phase arrest in PDAC cells [71]. Visudyne did not show any significant change in the levels of these mitotic proteins. Analysis using RalB antibodies showed that fendiline at 15 µM shows a slight reduction in RalB levels but the combinatorial treatment had no additional effect on the levels of this Ras family member (data not shown). The blots were re-probed with GAPDH for normalization of proteins on the blot.

All together these data suggest that the treatment strategies described here would be effective in overcoming various signaling pathways associated with PDAC growth, such as proliferation, survival, anchorage independent growth, self-renewal and drug resistance associated with pancreatic cancer cells, and would be of interest to determine if they elicit similar effects in vivo.

## 3. Discussion

PDAC is infamous for its aggressiveness, drug resistance and recurrence and currently there are no effective therapies to manage the disease. Though several drug combinations have been used to combat the disease, development of resistance and severe toxicity dampen their potential use. Under this circumstance, repurposing of any established drugs that are clinically proven to show very low to no toxicity in humans to be used in combinatorial therapies for PDACs appears to be an attractive option. Fendiline has been approved for treatment of angina in patients, with very little side effects. Our published studies and the results presented here suggest that the addition of fendiline to known chemotherapeutic agents such as gemcitabine, the YAP inhibitor visudyne or the c-Met and microtubule-targeting tivantinib, would ideally increase their cytotoxic effects. Though relatively high doses of fendiline are required to bring about strong inhibition of survival and oncogenic signaling, the combinations containing fendiline appear to bring down the amount required to get better effects, displaying its potential therapeutic value. 

An analysis of the molecular mechanisms targeted by these drugs reveal that they interfere with multiple signaling pathways associated with PDAC aggressiveness. One of the major genetic alterations seen in PDACs is the oncogenic mutation of K-Ras, which act through multiple downstream signaling pathways to promote tumor initiation and progression. We found that Akt and ERK activation are interrupted in PDAC cells treated with combinations of fendiline with other agents. Similarly, Ras-Ral axis is known to promote PDAC growth and co-treatments reduced the expression Ral A and B in the PDAC cells, with stronger effects on RalB. It has been shown that the Ral effector pathway, and not the canonical Ras-Raf-ERK pathway, is important for the K-Ras-dependent invadopodium formation, invasion and ECM remodeling [20]. Studies have also shown that the EMT markers, Slug and Snail, play an important role in PDAC invasiveness and fibrosis, respectively [72]. Western blot analysis of cells treated with fendiline alone or along with the chemotherapeutic agents mentioned here showed a marked reduction in their expression, especially slug, which was suppressed completely.

It has been shown that the oncogenic c-Myc is activated in PDAC and it serves as a master regulator of several cellular functions [73]. Thus, downregulation of c-Myc would be beneficial to the patients and may enhance their response to established chemotherapeutic agents. PDAC cells treated with fendiline and the pharmacological drugs used here showed a strong reduction in c-Myc levels and suggest that combinatorial treatment with fendiline would enhance the effectiveness of the established drugs in overcoming the Myc-dependent signaling.

Furthermore, studies have suggested that therapeutic agents would be more effective if Src inhibition is combined with treatment strategy based on the knowledge that Src confers chemo-resistance to PDAC. Src inhibition has been shown to reverse the 5-FU-associated chemoresistance by lowering the expression of thymidylate synthase (TS) [74]. Our finding that fendiline interferes with Src activation, by itself and in combination, thus suggests that combinatorial treatment with this calcium channel inhibitor would increase the efficacy of established therapies. Similarly, overexpression of Id1 has been associated with low survival in PDAC patients and Src is known to act upstream of Id1 to induce its expression. Similar to VEGF, Id1 has been shown to enhance tumor angiogenesis and microvessel density [23]. Furthermore, Id1 is known to enhance cell proliferation, DNA synthesis, migration and invasion of various cancer cells. We show that Id1 expression is reduced in cells treated with fendiline alone or in combination, whether this occurs downstream of Src needs to be determined. In addition, fendiline or visudyne alone and fendiline in combination with other agents significantly reduced the self-renewal capacity of cancer stem-like cells, implying that these agents could be effective in overcoming cancer recurrence and perhaps drug resistance in PDACs.

Taken together, our data show that combinatorial treatment of PDAC cells with fendiline and gemcitabine, tivantinib or visudyne effectively inhibits various oncogenic signaling mechanisms and suggest that these combinations would be effective in overcoming growth and metastatic characteristics along with drug resistance and recurrence observed in PDACs. Further testing of these combinations in normal human pancreatic ductal epithelial cells (HPDE6e7) cells and animal models is necessary to determine their safety and effectiveness in vivo and to establish if fendiline can be repurposed for better management of PDACs.

## 4. Materials and Methods 

### 4.1. Materials

DMEM containing 4.5 g/L Glucose, l-Glutamine and sodium pyruvate was from Corning, Penicillin/Streptomycin (100×) and Trypsin were from Thermo Fisher (Waltham, MA, USA), Fetal Bovine Serum was from VWR (Radnor, PA, USA), 40% Acrylamide solution and ECL solutions were from Thermo Fisher, TUNEL assay kit, Thiazolyl Blue Tetrazolium Bromide (MTT) and visudyne (Verteporfin) were from Sigma (St. Louis, MO, USA), tivantinib (ARQ197) was from ActiveBiochem (Hong Kong, China) or from Moffitt Chemistry Core (Moffitt Cancer Center and Research Institute), antibodies to Phospho-Src (Cell Signaling Technology Cat# 2101, RRID: AB_331697), Phospho-Histone H3 (*Cell Signaling Technology* Cat# 9701, *RRID*: AB_331535), Phospho-Aurora Kinase ABC (Cell Signaling Technology Cat# 2914, RRID: AB_2061631), RalB (Cell Signaling Technology Cat# 3523, RRID: AB_2176036), Slug (Cell Signaling Technology Cat# 9585, RRID:AB_2239535), Snail (Cell Signaling Technology Cat# 3879, RRID: AB_2255011), cleaved PARP (Cell Signaling Technology Cat# 9541, RRID: AB_331426), YAP1 (Cell Signaling Technology Cat# 8418, RRID: AB_10950494), Phospho-ERK (Cell Signaling Technology Cat# 4377, RRID: AB_331775) and Phospho-Akt (Cell Signaling Technology Cat# 4060, RRID:AB_2315049) were from Cell Signaling (Danvers, MA, USA), GAPDH (Sigma-Aldrich Cat# G8795, RRID: AB_1078991) and actin (Sigma-Aldrich Cat# A2228, RRID: AB_476697) antibodies were from Sigma, Id1 (Santa Cruz Biotechnology Cat# sc-488, RRID: AB_631701) and Cyclin D1 (Santa Cruz Biotechnology Cat# sc-753, RRID: AB_2070433) were from Santa Cruz Biotechnology (Dallas, TX, USA) and RalA (BD Biosciences Cat# 610221, RRID: AB_397618) was from BD Biosciences (San Jose, CA, USA).

### 4.2. Cell Lines

Panc1 (ATCC Cat# CRL-1469, RRID: CVCL_0480) and MiaPaCa2 (ATCC Cat# CRL-1420, RRID: CVCL_0428) cell lines used in this study were obtained from ATCC (Manassas, VA, USA); CD18/HPAF (abbreviated as CD18) cells were a kind gift from S. Batra [75,76]. Both Panc1 and MiaPaCa2 cells display mesenchymal characteristics and CD18 is epithelial in nature. These cells are maintained in DMEM supplemented with 10% Fetal Bovine Serum and 100 µg/mL Penicillin/Streptomycin.

### 4.3. MTT Assay

To measure cell viability, the MTT assay was conducted following established protocols [40,53]. 2500 cells were seeded per well in a 96 well plate and allowed to grow overnight. On the following day, media was removed and cells were treated with 100 μL of DMEM medium containing indicated concentrations of fendiline, visudyne or tivantinib alone or in combinations for the indicated periods of time. At the end of the incubation, 10 μL of 5mg/mL MTT was added to each well and incubated for 1 h. The media was removed, and purple formazan crystals formed in viable cells were solubilized in 100 μL of DMSO and absorbance was measured at 540 nm using a micro-plate reader. Higher absorbance correlates with more cell viability.

### 4.4. Soft Agar Colony Formation Assay

To determine whether the drugs individually or in combination inhibits anchorage-independent growth of cancer cells soft agar assays were conducted following our established protocols [40,53]. Briefly, 0.6% sterile agar in growth medium was added to 12-well plate and allowed to solidify at room temperature for 30 minutes to form the base layer. Subsequently, one ml of 0.3% Agar in growth medium, containing 2500 (for 24 well plates) or 5000 (12 well plates) CD18, MiaPaCa2 or Panc1 cells, with or without the indicated drugs was layered on top of the base layer. Plates were incubated in the CO_2_ incubator, and supplemented with 150 µL medium containing the drug or the vehicle (DMSO) once a week. After 14–21 days colonies were stained using MTT solution (1 mg/mL). Images of the colonies were taken and were quantified using the ImageJ image processing and analysis tool.

### 4.5. Sample Preparation

250,000 cells were plated in 60 mm dishes, allowed to grow overnight and treated with DMSO (vehicle), or indicated concentrations of fendiline (5 μM, 10 μM or 15 μM as indicated in figures), visudyne (1 µM), tivantinib (1.0 μM or 2.0 µM), or gemcitabine (1.0 µM), alone or in combination with fendiline, for 24 h. At the end of the treatment, cells were scraped into the medium, spun down at 1500 rpm for 5 min, washed twice with cold PBS and the cell lysates were prepared [77]. Lysates were cleared by centrifugation at 13,000 rpm for 15 min and equal amounts of proteins from the lysates were used for SDS-PAGE and western blot analysis.

### 4.6. SDS-PAGE and Western Blotting

For the SDS PAGE gels, 1.5 mm thick 12% Acrylamide gels were prepared. Equal amounts of proteins were loaded on the gel and were run at 90 V for approximately 2 h using the Bio-Rad Mini Protean Tetra System. Proteins were transferred onto 0.2 μm Nitrocellulose membrane for 30 min using the Trans-Blot Turbo Transfer System or for 1 h using the XCell II blot module transfer system from Invitrogen. Blots were then stained with Ponceau S solution (Sigma) for rapid staining of protein bands and blocked in 5% milk in TBS (blocking solution) for 1 h on a rocker at room temperature to prevent non-specific binding. After that, membranes were washed with PBS 3 times for 5 min each and were incubated with the antibodies indicated for overnight at 4 °C. At the end of the incubation, the blots were washed and incubated with anti-rabbit or anti-mouse HRP-conjugated secondary antibodies for one hour at room temperature, washed and developed using Pierce Super Signal ECL solution [40,53].

### 4.7. Sphere Formation Assay

To determine the self-renewal capacity of the cancer cells we conducted sphere formation assays following established protocols [55,78,79]. 1000 CD18 cells were plated per well in a 96 well ultra low attachment Corning plate (Fisher Scientific, Thermo Fisher) in serum-free DMEM/F12 medium supplemented with 1X-N2 supplement (Thermo Fisher,), 10 ng/mL EGF and 10 ng/mL bFGF (Sigma), with or without the indicated drugs. The spheres were allowed to grow for 10 days in the CO_2_ incubator and were imaged using an EVOS Phase Contrast inverted microscope. 

### 4.8. Migration (Wound Healing) Assay

Panc1 cells were plated onto a 12 well plate and incubated until the cells reached full confluence. A scratch was made along the diameter of each well using a 200 μL pipet tip [40]. Floating cells were removed by washing twice with DPBS and growth medium containing the indicated drugs were added to the wells. Images were taken immediately after the wounds were made and at 12 and 24 h afterwards using an EVOS Phase Contrast inverted microscope. The percentage of wound closure was calculated using the ImageJ image analysis tool.

### 4.9. Boyden Chamber Assay

Panc1 cells were pre-treated with the indicated drugs for one hour and 8000 cells were plated on to the Boyden chamber insert (Fisher Scientific, Thermo Fisher) kept in a 24-well dish containing DMEM with 20% serum and the drug. Cells were allowed to migrate for 4–6 h and fixed with paraformaldehyde, non-migrated cells from the top surface of the inserts were removed using a Q-tip, migrated cells were stained using Hoechst and analyzed on an EVOS inverted microscope.

### 4.10. TUNEL Assay

Panc1 cells cultured in 8-chamber slides were treated with indicated concentrations of gemcitabine, visudyne or tivantinib alone or in combination with fendiline and analyzed using the TUNEL assay kit (cat # 11684795910, In situ cell death detection, fluorescein, originally from Roche and currently from Signma Aldrich), following the instructions provided by the manufacturer. TUNEL positive cells were detected using the EVOS inverted microscope.

### 4.11. Statistical Analysis

All the experiments were conducted at least three independent times, cytotoxicity analysis, migration assay, soft agar assay and self renewal were conducted at least 3 times in triplicates and statistical analysis was conducted using Student’s *t* test.

## Supplementary Materials

Supplementary materials can be found at https://www.mdpi.com/1422-0067/20/10/2423/s1.

## Abbreviations

PDAC	Pancreatic ductal adenocarcinoma
YAP1	Yes-associated protein 1
Id1	Inhibitor of differentiation 1
PARP GEM	Poly ADP ribose polymerase Genetically engineered mouse
