# Supplementary material for: Fendiline Enhances the Cytotoxic Effects of Therapeutic Agents on PDAC Cells by Inhibiting Tumor-Promoting Signaling Events: A Potential Strategy to Combat PDAC
Source: Int J Mol Sci. 2019 May 16;20(10):2423. doi: 10.3390/ijms20102423 (PMC6567171; doi:10.3390/ijms20102423)
Supplement: Supplementary file 1 [file ijms-20-02423-s001.pdf]

Supplementary files:

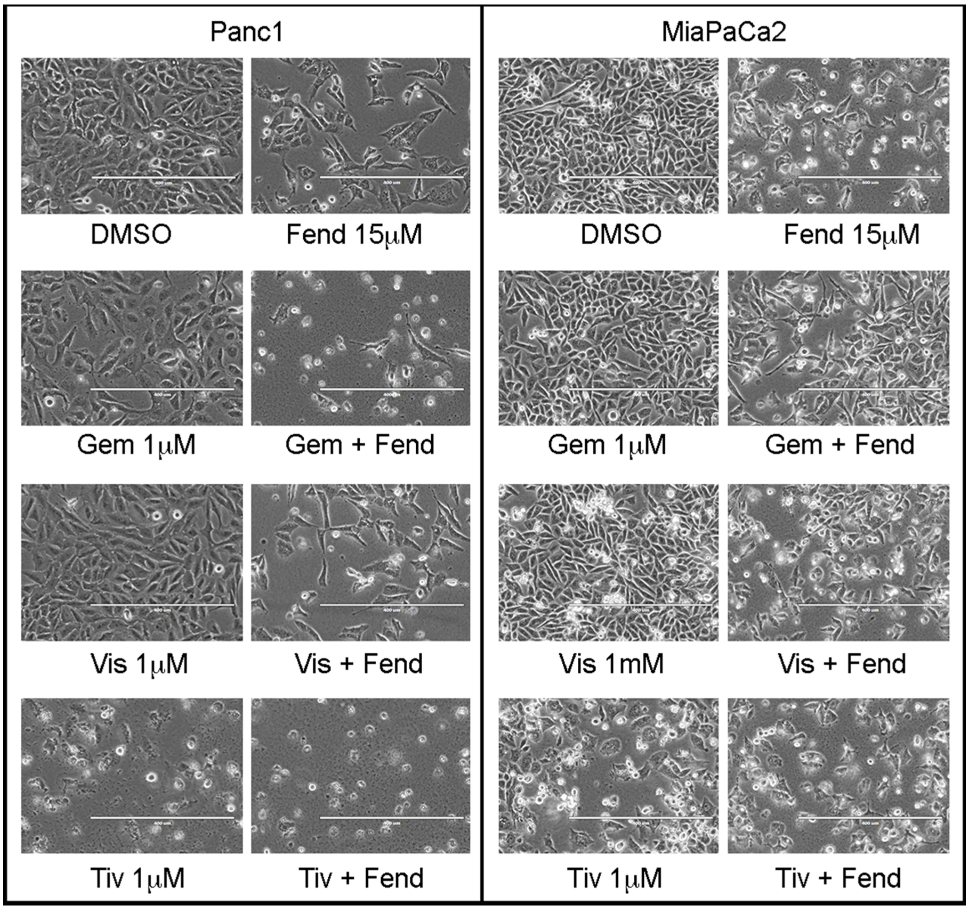

Supplementary figure 1: Morphology of Panc1 and MiaPaCa2 cells treated with fendiline and the various drugs alone or in combination for 48 hours, images were taken using an EVOS inverted microscope. Cells co-treated with tivantinib and fendiline showed more apoptotic and mitotic cells whereas the other agents appeared to reduce the growth of the cells when the treatment was done in combination with fendiline. Magnification 10X.

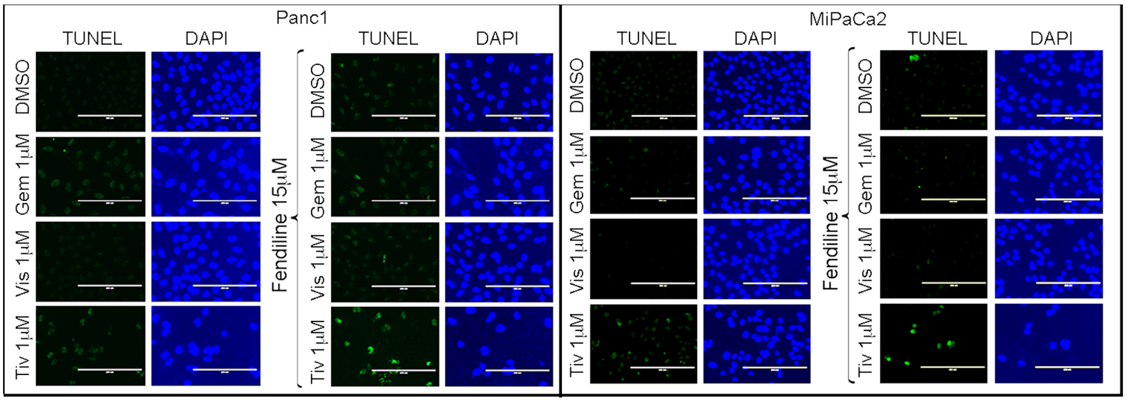

Supplementary Figure 2: MiaPaCa2 and Panc1 cells were treated with the indicated drugs for 24 hours and TUNEL positivity was determined using the *in situ* cell death detection kit (fluorescein). We observed TUNEL positivity mainly in cells co-treated with tivantinib and fendiline. Magnification 10X.
